# The Effect of Whitening Toothpastes on the Color Stability of a Smart Monochromatic Composite Resin

**DOI:** 10.7759/cureus.46225

**Published:** 2023-09-29

**Authors:** Begum Tavas, Ozge Celiksoz, Hatice Tepe, Sanem Ozaslan, Batu Can Yaman

**Affiliations:** 1 Department of Restorative Dentistry, Eskisehir Osmangazi University Faculty of Dentistry, Eskişehir, TUR

**Keywords:** monoshade composite resin, sensodyne 2 in 1 whitening, sensodyne fresh mint, colgate optic white with charcoal, omnichroma, composite resin, discoloration, whitening toothpaste

## Abstract

Objective: The aim of this study was to evaluate the color stability of smart monochromatic composite resin after coloring with coffee solution, thermal aging and brushing with four different kinds of toothpaste.

Materials and methods: According to the manufacturer's instructions, 40 smart monochromatic composite resin (Omnichroma, Tokuyama Dental, Japan) specimens were prepared with a thickness of 2 mm and a diameter of 10 mm. The samples were divided into four groups. The first group (SFM) was brushed with Sensodyne Fresh Mint (Sensodyne GSK, UK), the second group (CW) with Colgate 2 in 1 Whitening (Colgate Palmolive, USA), the third group (OW) with Opalescence Whitening (Ultradent Products, Inc., USA), the fourth group (COW) with Colgate Optic White With Charcoal (Colgate Palmolive, USA). At time point t_0_, no brushing and thermal cycles were performed. For time point t_1_, simulations corresponding to 10 days of staining, thermal aging and brushing were performed. For time point t_2_, simulations corresponding to one year of staining, thermal aging and brushing were performed. The color of all specimens was measured at t_0_, t_1_ and t_2_ with a spectrophotometer. To examine the color change, ΔE_00 _values were calculated with the CIEDE 2000 color system. Shapiro Wilk, Kolmogorov Smirnov, Wilcoxon Signed Rank, Kruskal Wallis, and Mann-Whitney U tests were used to analyze the data.

Results: According to the results of the intergroup comparison, there is no statistically significant difference between the groups in ΔE_00_(t_0_-t_1_) values in terms of t_0_-t_1 _time period measurement (p>0.05). There is a statistically significant difference between the groups in ΔE_00_(t_1_-t_2_)values in terms of t_1_-t_2_ time period measurement (p<0.05). The COW group had the lowest ΔE_00_(t_1_-t_2_) value and the OW group had the highest ΔE_00_(t_1_-t_2_) value. There is a statistically significant difference between the groups in ΔE_00_ (t_0_-t_2_) values in terms of t_0_-t_2_ time period measurement (p<0.05). The COW group had the lowest ΔE_00_(t_0_-t_2_) value and the OW group had the highest ΔE_00_(t_0_-t_2_) value.

Conclusion: The whitening efficacy of different kinds of toothpaste whitening mechanisms may differ from each other. Toothpastes also show whitening on composite resins. The lowest discoloration was observed in the group brushed with toothpaste containing activated charcoal and blue covarine. The efficacy of whitening toothpastes should be supported by in vivo studies.

## Introduction

Nowadays, people's aesthetic expectations are increasing day by day. Although dental aesthetics is related to many factors, it has been found that individuals care more about the color of their teeth than other factors [1]. The need for teeth whitening to remove discoloration has increased and as a result, non-invasive whitening products have been developed [2,3]. Apart from the whitening procedures that dentists can apply in their clinics, whitening toothpastes are also one of the whitening products that patients can apply on their own. The whitening effectiveness of toothpastes is provided by mechanical, chemical and optical methods [4,5]. The abrasives in toothpastes provide a mechanical whitening effect. These abrasives are hydrated silica, silica, calcium carbonate, calcium pyrophosphate, dicalcium phosphate dihydrate, perlite, alumina and sodium bicarbonate. Abrasives are effective only on external coloration and on the areas where the brush comes into contact. Abrasives can cause abrasion and deterioration of the tooth structure. Therefore, chemical whitening agents are added to whitening toothpastes [6,7]. Chemical whitening agents are hydrogen peroxide, calcium peroxide, sodium citrate, sodium pyrophosphate, sodium tripolyphosphate and sodium hexametaphosphate [4]. With the development of color science, the addition of blue covarin to toothpastes has brought a different approach to whitening and toothpastes with optical whitening have been introduced to the market. Blue covarine is deposited on the tooth surface and improves the optical properties of teeth. In a study concluded that toothpastes containing blue covarine or a combination of blue covarine and FD&C blue No. 1 significantly improved the whiteness of teeth immediately after brushing in both in vitro and clinical settings [8].

Recently, whitening toothpastes containing activated charcoal have become popular products, aiming to whiten teeth by adhering to discoloration on the tooth surface and removing stains [7]. Toothpastes containing activated charcoal affect tooth color by adsorbing the stains on the teeth into the pores due to the pigment, chromophore and nanocrystalline carbon structure it contains. It is recommended by dentists to patients to increase maintenance after teeth whitening treatment. However, since activated charcoal has a high absorption capacity, it negatively affects the remineralization of dental tissues by reducing fluoride ions in toothpastes [9,10].

It has been reported that tooth brushing also affects the surface properties of composite resins. The polymer matrix of the composite resin is eroded by brushing and the inorganic structure is exposed. Therefore, the restoration surface may become rough and its sensitivity to coloration may increase [11]. However, it has also been reported that tooth brushing may reduce the amount of color change by separating the coloring pigments accumulated in the recesses of the restorative materials from the surface [12].

Color stability is one of the most important factors affecting the life of composite resins. A rough surface structure can cause staining and discoloration. Therefore, it is very important to clarify the effects of whitening toothpastes on restorative materials [2,13].

The aim of this study was to evaluate the color stability of monochromatic composite resin after coloring with coffee solution, thermal aging and brushing with four different toothpastes.

The null hypothesis formed for this aim is “The effectiveness of different toothpastes on the color stability of the smart monochromatic composite resin will not be different.”

## Materials and methods

**Figure 1:**
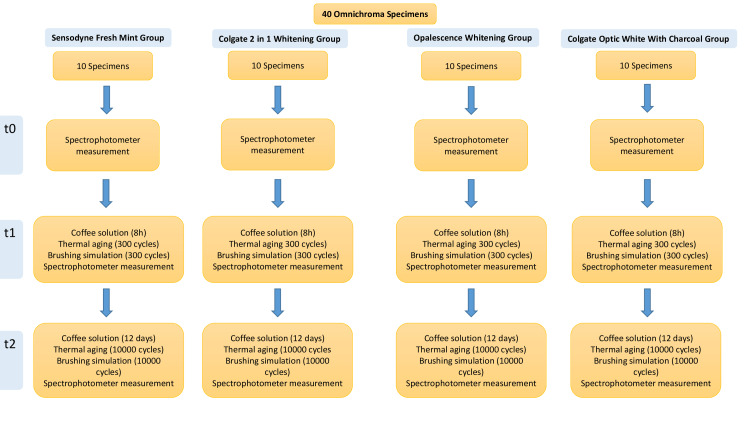
Work Flow Chart

The name, brand, manufacturer, table of contents, usage procedures and lot numbers of the materials used in the study are shown in Table 1. The work flow chart is shown in Figure 1.

**Table 1:** Materials Used in the Study

Material	Brand	Manufacturer	Table of Contents	Usage Procedures
Composite Resin (LOT: 04EZ0)	Omnichroma	Tokuyama Dental, Japan	79 wt% (68 by volume) spherical silica-zirconia fillers, 1,6-bisUDMA, TEGDMA, Mequinol, Dibutyl hydroxyl toluene and UV absorber	20s with 600 mW/cm^2^ light power with halogen or LED light device at 400-500 nm wavelength
Toothpaste (RDA: 90) (LOT: 31803KWA)	Sensodyne Fresh Mint	Sensodyne GSK, United Kingdom	Aqua, Sorbitol, Hydrated Silica, Glycerin, Potassium Nitrate, Cocamidopropyl Betaine, Aroma, Xanthan Gum, Titanium Dioxide, Sodium Fluoride, Sodium Saccharin, Sodium Hydroxide, Sucralose, Limonene. Sodium Fluoride 0.315% w/w (1450 ppm fluoride)	
Toothpaste (RDA: 200) (LOT:1027MX111A)	Colgate 2in 1 Whitening	Colgate Palmolive, ABD	Sodium Fluoride (0.24%) (0.14% w/v Fluoride Ion). Inactive Ingredients: Water, Sorbitol, Glycerin, Hydrated Silica, Sodium Lauryl Sulfate, Flavor, Tetrasodium Pyrophosphate, Sodium Saccharin, Cocamidopropyl Betaine, Cellulose Gum, Xanthan Gum, Titanium Dioxide	
Toothpaste (RDA: 66) (LOT: BN3MV)	Opalescence Whitening	Ultradent Products, Inc., USA	Sodium Fluoride 0.25% w/w, Glycerin, Water (aqua), Silica, Sorbitol, Xylitol, Flavor, Poloxamer, Sodium Lauryl Sulfate, Carbomer, FD&C Blue#1 (Cl 42090), FD&C Yellow#5 (Cl 19140), Sodium Benzoate, Sodium Hydroxide, Sucralose, Xanthan Gum	
Toothpaste (RDA: 76) (LOT: 261122PL1133)	Colgate Optic White With Charcoal	Colgate Palmolive, USA	Water, Hydrated Silica, Sorbitol, Glycerin, PEG-12, Pentasodium Triphosphate, Tetrapotassium Pyrophosphate, Sodium Lauryl Sulfate, Taste, Cellulose Gum, Cocamidopropyl Betaine, Sodium Saccharin, Xanthan Gum, Coal Powder, Sodium Hydroxide, Blue 1, Red 40, Titanium Dioxide	
Finishing and Polishing Spiral (LOT: 436372)	Twist Diacomp Plus	Eve Earnst Vetter GmbH, Germany	Diamond-impregnated flexible spirals	Front polishing 20 sec 8000 rpm Polishing spiral 2 sec 8000 rpm
Polishing Paste (LOT:191085)	SDI Polishing Paste	SDI Limited, Australia	Glycerin, aluminum oxide	30s at low speed and pressure
Polishing Brush (LOT: BHZHT)	Jiffy goat hair brush	Ultradent Product, USA		30s at low speed and pressure
Toothbrush	Colgate Extra Clean 1+1	Colgate Palmolive, USA		
L.E.D. Light Source	Dentsply Sirona SmartLite Focus	Dentsply Sirona, USA		

Preparation of specimens

In the present study, a total of 40 composite resin specimens (Omnichroma, Tokuyama Dental, Japan), 10 in each group, were prepared using a 10x2 mm silicone mold. The composite resins were condensed in the silicone mold and polymerization was performed on a mylar strip and a transparent slide using a SmartLite Focus (Dentsply, USA) LED light device for 20 seconds. After the transparent slide was removed, an additional 20s of polymerization was performed. The specimens were removed from the silicone mold and the back surfaces were also polymerized for 20 seconds [14]. All specimens were kept in distilled water at 37°C in an oven for 24 hours. All specimens were abraded with 600 grit (Metaserv 250, Buehler, Germany) sandpaper under water for 20 seconds. All specimens were pre-polished (pink) and polished (gray) with a polishing spiral (Twist Diacomp Plus, Eve Earnst Vetter GmbH, Germany) for 20 seconds each under water cooling at a speed of 8000 rpm according to the manufacturer's instructions. Then all specimens were polished with a paste (Polishing paste, SDI Limited, Australia) and a goat hair brush (Jiffy, Ultradent Product, USA) at low speed and low pressure for 30 seconds according to the manufacturer's instructions.

Formation of groups

The specimens were divided into four groups: SFM (Sendoyne Fresh Mint, Sensodyne GSK, UK), CW (Colgate 2 in 1 Whitening, Colgate Palmolive, USA), OW (Opalescence Whitening, Ultradent Products, Inc., USA), COW (Colgate Optic White With Charcoal, Colgate Palmolive, USA).

Staining procedure

For time t_0_, not kept in coffee solution. For time t_1_, they were kept in coffee solution for 8 hours, corresponding to 10 days of coloration. For time t_2_, the specimens were kept in a coffee solution for 12 days, corresponding to one year of coloring. The coffee solution was prepared using 2 g soluble coffee (Nescafe Gold, Nestle, Switzerland), 200 ml boiled water and kept at 37⁰C for a while. Eppendorf tubes of 1.5 mm in size were used to hold the specimens separately in the solutions. The tubes in which the solution and specimen were placed were kept in an oven (Binder KB115 & Nuve ES252, Radobio Scientific CO., China) at 37⁰C for 8 hours in order to be suitable for oral conditions. The solutions in the tubes were changed daily.

Thermal aging procedure

No thermal aging was applied for time t_0_. For time t_1 _300 thermal cycles corresponding to 10 days of thermal aging were applied. For time t_2_ 10000 thermal cycles corresponding to one year of thermal aging were applied [15]. the specimens were subjected to at 5-55°C (±2°C) with a transfer time of 10 seconds and a waiting time of 30 seconds in the tank, respectively, in an Esetron thermal cycling device (Mod Dental, Esetron Smart Robotechnologies, Turkey).

Brushing simulation procedure

No brushing simulation was applied for time t_0_. For time t_1_ 300 cycles of brushing simulation corresponding to 10 days of brushing. For time t_2_ 10000 cycles of brushing simulation corresponding to one year of brushing were applied [16]. Mod Dental MTB 100 (Mod Dental, Esetron Smart Robotechnologies, Turkey) brushing simulator was used in the study. A paste diluted 1/3 by volume was used. The device completed the simulation under a 250 g load, in circular brushing mode, with a movement diameter of 10 mm.

Color measurement

The color values of all specimens were measured at the end of time periods t_0_, t_1_ and t_2_ with a VITA Easyshade V (VITA Zahnfabrik, Bad Säckingen, Germany) on a gray background in 3-point measurement mode and the instrument was recalibrated after each measurement. The instrument was calibrated before all measurements. The average of the L*a*b* values measured at each of the 3 points was calculated to obtain the average value for each specimen. Then, from this average value, the ΔE00 value showing the color change with the CIEDE2000 color system was calculated from the online color calculator (www.colormine.org).

Statistical analysis

Within the scope of the research, the Statistical Package for the Social Sciences (IBM SPSS Statistics for Windows, IBM Corp., Version 26.0, Armonk, NY) statistical program was used. Shapiro Wilk and Kolmogorov Smirnov tests were used to analyze whether the data evaluated within the scope of the study were suitable for normal distribution. According to the results obtained, the p-value for the data was found to be below 0.05 and it was also concluded that the specimen size was suitable for non-parametric test criteria. Wilcoxon Signed Rank test was used for the general comparison of materials in terms of time periods in ΔE00 measurements. Kruskal-Wallis test was used for the comparison of ΔE00 measurements between groups in the measured time period. Mann-Whitney U test was applied for pairwise comparisons between materials in measurements determined to be significantly different. The significance level was set as 0.05 within the scope of the study.

## Results

The group comparison of ΔE00 values is in Table 2. A comparison of ΔE00 measurements between time periods in the groups is shown in Table 3.

**Table 2:** Group Comparison of ΔE00 Values Exponential letters are used for comparison between groups. There is no difference between groups with the same letter. SFM: Sensodyne Fresh Mint, CW: Colgate 2 in 1 Whitening, OW: Opalescence Whitening, COW: Colgate Optic White With Charcoal

		N	Mean	Mean Rank	S.S.	p
t_0_-t_1_	SFM	10	1.14	14.17	0.68	0.326
	CW	10	1.53	19.17	0.86	
	OW	10	1.40	17.44	0.56	
	COW	10	1.87	23.22	0.88	
	Total	40	1.48		0.77	
t_1_-t_2_	SFM	10	6.05^a^	17.39	1.45	0.005*
	CW	10	6.57^a^	21.28	0.91	
	OW	10	7.13^b^	26.22	0.68	
	COW	10	4.95^c^	9.11	1.39	
	Total	40	6.17		1.37	
t_0_-t_2_	SFM	10	6.77^a^	17.67	1.42	0.003*
	CW	10	7.39^b^	22.67	0.49	
	OW	10	7.59^b^	25.44	0.55	
	COW	10	6.04^c^	8.22	1.07	
	Total	40	6.95		1.11	

**Table 3:** Comparison of ΔE00 Measurements between Time Periods in Groups SFM; ΔE00 (t_0_-t_1_)-(t_1_-t_2_) p=0.001, ΔE00 (t_0_-t_1_)-(t_0_-t_2_) p=0.001, ΔE00 (t_0_-t_2_)-(t_1_-t_2_) p=0.003 CW; ΔE00 (t_0_-t_1_)-(t_1_-t_2_) p=0.001, ΔE00 (t_0_-t_1_)-(t_0_-t_2_) p=0.001, ΔE00 (t_0_-t_2_)-(t_1_-t_2_) p=0.002 OW; ΔE00 (t_0_-t_1_)-(t_1_-t_2_) p=0.001, ΔE00 (t_0_-t_1_)-(t_0_-t_2_) p=0.001, ΔE00 (t_0_-t_2_)-(t_1_-t_2_) p=0.008 COW; ΔE00 (t_0_-t_1_)-(t_1_-t_2_) p=0.001, ΔE00 (t_0_-t_1_)-(t_0_-t_2_) p=0.001, ΔE00 (t_0_-t_2_)-(t_1_-t_2_) p=0.006 SFM: Sensodyne Fresh Mint, CW: Colgate 2 in 1 Whitening, OW: Opalescence Whitening, COW: Colgate Optic White With Charcoal

		n	Mean	S.D.
SFM	t_0_-t_1_	10	1.14	0.68
	t_1_-t_2_	10	6.05	1.45
	t_0_-t_2_	10	6.77	1.42
CW	t_0_-t_1_	10	1.53	0.86
	t_1_-t_2_	10	6.57	0.91
	t_0_-t_2_	10	7.39	0.49
OW	t_0_-t_1_	10	1.40	0.56
	t_1_-t_2_	10	7.13	0.68
	t_0_-t_2_	10	7.59	0.55
COW	t_0_-t_1_	10	1.87	0.88
	t_1_-t_2_	10	4.95	1.39
	t_0_-t_2_	10	6.04	1.07

According to the results of the intergroup comparison, there is no statistically significant difference between the groups in ΔE00(t_0_-t_1_) values in terms of t_0_-t_1_ time period measurement (p>0.05). However, the highest ΔE00 value was observed in the COW group and the lowest ΔE00 value was observed in the SFM group.

According to the results of the intergroup comparison, there is a statistically significant difference between the groups in ΔE00(t_1_-t_2_) values in terms of t_1_-t_2_ time period measurement (p<0.05). While SFM and CW groups were similar to each other, there was a difference between all other pairwise comparisons. The COW group had the lowest ΔE00 value and OW group had the highest ΔE00 value.

According to the results of the intergroup comparison, there is a statistically significant difference between the groups in ΔE00(t_0_-t_2_) values in terms of t_0_-t_2_ time period measurement (p<0.05). While OW and CW groups were similar to each other, there was a difference between all other pairwise comparisons. The COWl group had the lowest ΔE00 value and OW group had the highest ΔE00 value.

## Discussion

The present study was conducted to evaluate the discoloration effect of different whitening toothpastes on composite resins colored with coffee solution and thermal aging. As a result of the study, the COW group had the lowest ΔE00 value and the OW group had the highest ΔE00 value. According to these results, the null hypothesis is rejected.

Smart monochromatic composite resins are preferred by dentists today because they reduce the need for different colored composite resins, minimize composite resin waste, shorten the time spent at the bedside and eliminate the need for color selection [17]. Omnichroma is a smart monochromatic composite resin. It provides color matching without any color pigment and contains 260 nm suprananospheric filler particles [18,19]. Omnichroma was used in our study, considering the need for further research on color stability.

Composite resins are colored as a result of intrinsic and extrinsic factors. Intrinsic factors include unreacted methacrylate groups, oxidation of the polymer matrix and amine accelerators, while extrinsic factors include absorption and adsorption of coloring agents [20]. In many studies examining the color change of composite resins, it has been shown that beverages such as coffee, tea and wine have an effect on color [12]. In the studies conducted, the drinking time of one cup of coffee was calculated as 15 minutes and an average daily consumption of 2-3 cups of coffee was reported [21]. Therefore, in our study, 12 days of kept-in coffee solution was performed for one year of coffee consumption.

Thermal cycling is one of the most common practices used to mimic physiological aging. It is a repeated cycle in hot and cold water tanks to reflect the thermal changes occurring in the oral cavity [22]. In one study, the lowest average intraoral temperature was 5⁰C and the highest was 55⁰C. Thermal cycling occurs on average 20-50 times a day, which corresponds to an average of 10,000 cycles per year [15]. According to studies, every 10,000 cycles performed in the brushing simulator corresponds to one year of brushing [16].

In dental studies, color change (ΔE) is often calculated with the CIEL*a*b* system. However, the CIEDE2000 color system developed by Munsell in 2001 was developed to calculate more accurate color change (ΔE00) by making modifications to the factors affecting the perception of the eye [23,24]. In line with this information, the CIEDE2000 color system was used to evaluate color change in our study.

In studies investigating discoloration, toothpastes were reported to cause discoloration [25]. The whitening efficacy of some whitening toothpastes has been reported to result from the removal of superficial stains as a result of their abrasive properties, as well as chemical whitening with peroxide or similar ingredients [26]. Detergents in toothpastes (e.g. sodium lauryl sulfate) and their pH values are also thought to affect the surface properties of restorative materials [27]. Some studies have shown that optical whitening toothpastes have a greater effect on discoloration than conventional toothpastes [28].

Relative dentin abrasivity (RDA) is a measurement of dentin abrasiveness. Abrasives can prevent extrinsic discoloration. The International Organization for Standardization (ISO) recommends that the RDA of toothpaste should not exceed 250, and the average RDA of whitening toothpastes is between 60 and 100 or higher than 100 [5,29]. De Moraes Rego Roselino et al. [28] evaluated the effect of whitening toothpastes on different composites in a clinical setting in an in situ study and reported that different abrasive toothpastes had an insignificant effect on the color stability of composites [30]. In a study examining the surface roughness and color change of mechanical brushing on two different composite resins, it was concluded that toothpaste abrasiveness had no effect on color [31]. In an in situ study examining the color stability of whitening toothpastes on different composite resins, it was found that pastes with different abrasiveness had no effect on color stability [30]. In three different studies investigating the effect of whitening toothpastes on the discoloration of composite resins, as a result of experiments without coloring and thermal aging; Al Shalan and Roopa [32,33] found a significant difference in the color change of composite resins, while Hashemikamangar et al. [14] toothpastes had no effect on the color of composite resins. Although the aging procedure was not applied in the mentioned studies, it can provide information about toothpaste comparison. No direct correlation could be established between the RDA values of the toothpastes used in our study and color change.

Toothpastes containing activated charcoal show significant whitening efficiency by absorbing coloring pigments, chromophores and stains thanks to the surface area of carbon. The high absorption capacity of activated charcoal also affects the concentration of fluorine and other active ions in the toothpaste. Because of this absorption capacity, toothpastes containing activated charcoal may be insufficient for remineralization of enamel. Therefore, starting to use activated charcoal toothpaste may increase the risk of caries in an individual who uses normal fluoride-containing toothpaste. Brooks et al. examined 50 toothpastes containing activated charcoal and found that only four of them contained fluoride [34]. Charcoal has also been described as abrasive to tooth and gum tissues. It was found that toothpastes containing charcoal, silica and hydrated silica can cause surface roughness higher than the roughness threshold value of 0.2 µm after tooth brushing [35-38]. However, studies on the effects of activated charcoal on human health are insufficient. The possible risks to human health of toothpastes containing activated charcoal are thought to be related to polyaromatic hydrocarbons and bentonite clay [34,38]. In a study, it was reported that more studies and scientific evidence are needed for the possible side effects of toothpastes containing activated charcoal [34]. Colgate Activated Charcoal toothpaste used in our study gave a significantly lower ΔE00 value than the other groups at t_1_-t_2_ and t_0_-t_2_ time periods and was found to have the highest whitening efficiency among the toothpastes used in our study.

Limitations

Only color change was evaluated in our study. Intraoral thermal changes and brushing simulation were simulated in the laboratory and the most important limitation of the study is the lack of in vivo environment. In addition, the lack of advanced measurements such as profilometry, scanning electron microscopy and atomic force microscopy are also limitations of the study.

## Conclusions

Whitening toothpastes are easily accessible to patients and cause discoloration on teeth and composite resin restorations. The whitening efficacy of toothpastes with different mechanisms may be different from each other. While the most effective whitening was achieved with Colgate Activated Charcoal toothpaste in our study, the efficacy and side effects of whitening toothpastes need to be supported by in vivo studies.

## References

[1] Dudea D, Lasserre JF, Alb C, Culic B, Pop Ciutrila IS, Colosi H (2012). Patients' perspective on dental aesthetics in a South-Eastern European community. J Dent.

[2] Yilmaz MN, Gul P, Unal M, Turgut G (2021). Effects of whitening toothpastes on the esthetic properties and surface roughness of a composite resin. J Oral Sci.

[3] Marshall K, Berry TG, Woolum J (2010). Tooth whitening: current status. Compend Contin Educ Dent.

[4] Joiner A (2010). Whitening toothpastes: a review of the literature. J Dent.

[5] Maldupa I, Brinkmane A, Rendeniece I, Mihailova A (2012). Evidence based toothpaste classification, according to certain characteristics of their chemical composition. Stomatologija.

[6] Vertuan M, de Souza BM, Machado PF, Mosquim V, Magalhães AC (2020). The effect of commercial whitening toothpastes on erosive dentin wear in vitro. Arch Oral Biol.

[7] Vaz VT, Jubilato DP, Oliveira MR, Bortolatto JF, Floros MC, Dantas AA, Oliveira Junior OB (2019). Whitening toothpaste containing activated charcoal, blue covarine, hydrogen peroxide or microbeads: which one is the most effective?. J Appl Oral Sci.

[8] Tao D, Sun JN, Wang X, Zhang Q, Naeeni MA, Philpotts CJ, Joiner A (2017). In vitro and clinical evaluation of optical tooth whitening toothpastes. J Dent.

[9] Dionysopoulos D, Papageorgiou S, Malletzidou L, Gerasimidou O, Tolidis K (2020). Effect of novel charcoal-containing whitening toothpaste and mouthwash on color change and surface morphology of enamel. J Conserv Dent.

[10] Ghajari MF, Shamsaei M, Galouyak MS, Basandeh K (2022). Evaluation of abrasion and whitening effect of toothpastes containing charcoal on primary teeth. Front Dent.

[11] Neme A, Frazier KB, Roeder L, Debner T (2002). Effect of prophylactic polishing protocols on the surface roughness of esthetic restorative materials. Operative dentistry.

[12] Bezgin T, Özer L, Tulga Öz F, Özkan P (2015). Effect of toothbrushing on color changes of esthetic restorative materials. J Esthet Restor Dent.

[13] Meyers IA, McQueen MJ, Harbrow D, Seymour GJ (2000). The surface effect of dentifrices. Aust Dent J.

[14] Hashemikamangar SS, Hoseinpour F, Kiomarsi N, Dehaki MG, Kharazifard MJ (2020). Effect of an optical whitening toothpaste on color stability of tooth-colored restorative materials. Eur J Dent.

[15] Gale M, Darvell B (1999). Thermal cycling procedures for laboratory testing of dental restorations. J Dent.

[16] Goldstein GR, Lerner T (1991). The effect of toothbrushing on a hybrid composite resin. J Prosthet Dent.

[17] AlHamdan EM, Bashiri A, Alnashmi F (2021). Evaluation of smart chromatic technology for a single-shade dental polymer resin: an in vitro study. Appl Sci.

[18] Pereira Sanchez N, Powers JM, Paravina RD (2019). Instrumental and visual evaluation of the color adjustment potential of resin composites. J Esthet Restor Dent.

[19] Ahmed MA, Jouhar R, Vohra F (2022). Effect of different pH beverages on the color stability of smart monochromatic composite. Appl Sci.

[20] Asmussen E, Hansen EK (1986). Surface discoloration of restorative resins in relation to surface softening and oral hygiene. Scand J Dent Res.

[21] Zajkani E, Abdoh Tabrizi M, Ghasemi A, Torabzade H, Kharazifard M (2013). Effect of staining solutions and repolishing on composite resin color change. J Iran Dent Assoc.

[22] Morresi AL, D'Amario M, Capogreco M, Gatto R, Marzo G, D'Arcangelo C, Monaco A (2014). Thermal cycling for restorative materials: does a standardized protocol exist in laboratory testing? A literature review. J Mech Behav Biomed Mater.

[23] Ghinea R, Pérez MM, Herrera LJ, Rivas MJ, Yebra A, Paravina RD (2010). Color difference thresholds in dental ceramics. J Dent.

[24] Çarıkçıoğlu B (2021). Effect of different other whitening products sold without prescription on the color changes of colored nano and micro hybrid composites. J Ataturk Univ Fac Dent.

[25] Joiner A, Philpotts CJ, Alonso C, Ashcroft AT, Sygrove NJ (2008). A novel optical approach to achieving tooth whitening. J Dent.

[26] Amaral CM, Rodrigues JA, Erhardt MC, Araujo MW, Marchi GM, Heymann HO, Pimenta LA (2006). Effect of whitening dentifrices on the superficial roughness of esthetic restorative materials. J Esthet Restor Dent.

[27] Addy M (2002). Dentine hypersensitivity: new perspectives on an old problem. Int Dent J.

[28] Torres CR, Perote LC, Gutierrez NC, Pucci CR, Borges AB (2013). Efficacy of mouth rinses and toothpaste on tooth whitening. Oper Dent.

[29] Patil P, Ankola A, Hebbal MI, Patil A (2015). Comparison of effectiveness of abrasive and enzymatic action of whitening toothpastes in removal of extrinsic stains-a clinical trial. Int J Dent Hyg.

[30] de Moraes Rego Roselino L, Tonani Torrieri R, Sbardelotto C (2019). Color stability and surface roughness of composite resins submitted to brushing with bleaching toothpastes: an in situ study. J Esthet Restor Dent.

[31] Roselino Lde M, Cruvinel DR, Chinelatti MA, Pires-de-Souza Fde C (2013). Effect of brushing and accelerated ageing on color stability and surface roughness of composites. J Dent.

[32] Roopa K, Basappa N, Prabhakar A, Raju O, Lamba G (2016). Effect of whitening dentifrice on micro hardness, colour stability and surface roughness of aesthetic restorative materials. J Clin Diagnostic Res.

[33] Al-Shalan T (2017). Effect of whitening toothpastes on color stability of different restorative materials. Int J Med Sci Clin Invent.

[34] Brooks JK, Bashirelahi N, Reynolds MA (2017). Charcoal and charcoal-based dentifrices: a literature review. J Am Dent Assoc.

[35] Pertiwi U, Eriwati YK, Irawan B (2017). Surface changes of enamel after brushing with charcoal toothpaste. J Phys Conf Ser.

[36] Değer C, Müjdeci A (2020). Whitening dentifrices: a review. Cyprus J Med Sci.

[37] Tembhurkar A, Dongre S (2006). Studies on fluoride removal using adsorption process. J Environ Sci Eng.

[38] Greenwall LH, Greenwall-Cohen J, Wilson NH (2019). Charcoal-containing dentifrices. Br Dent J.

